# Experimental paradigm to test the effects of providing social support: study protocol of the PROSPECT trial (Study 2)

**DOI:** 10.1186/s40359-024-02319-y

**Published:** 2025-01-27

**Authors:** Vivien Hajak, Simone Grimm, Ewa Gruszczyńska, Aleksandra Kroemeke, Natalia Józefacka, Lisa Marie Warner

**Affiliations:** 1 Department of Psychology, MSB Medical School Berlin, Rüdesheimer Str. 50, Berlin, 14197 Germany; 2 SWPS University, Institute of Psychology, Health & Coping Research Group, Warsaw, Poland

**Keywords:** Support provision, Helping, Prosocial behaviour, Self-determination theory, Affect, Cortisol, Blood pressure, Heart rate, Empathy

## Abstract

**Background:**

A growing body of research suggests that the provision of social support can have benefits not only for the recipients but also for the provider. Although initial evidence for affective, self-evaluative and physiological outcomes has been established, the beneficial effects of support provision do not occur consistently across all support interactions, and some interactions may even have detrimental effects on providers. The aim of our experimental paradigm is to enable researchers to test the conditions under which the provision of social support to dyadic partners affects affective, self-evaluative, physiological, and relationship outcomes for the provider. In line with self-determination theory, it is proposed that the provision of support is only beneficial to the provider if it satisfies the three basic psychological needs of autonomy, competence and relatedness. The paradigm allows for the manipulation of the provider's levels of competence (feedback on the effectiveness of their support to the other person) and relatedness (feedback on the alleged level of relatedness perceived by the partner person following the provision of support).

**Methods:**

A priori power analyses resulted in a planned sample size of 250 participants randomized to four conditions: 1) no support provision, 2) support provision without feedback, 3) support provision with feedback on competence, 4) support provision with feedback on relatedness. Primary outcomes are immediate physiological (saliva cortisol, heart rate, heart rate variability, blood pressure), affective (positive and negative affect, anxiety), self-evaluative (e.g., self-esteem) and relationship outcomes. Generalized linear models will be used to compare the four conditions.

**Discussion:**

In a controlled laboratory experiment, this new experimental paradigm manipulates the conditions under which social support is provided. Insights into the conditions under which the provision of social support is detrimental or beneficial to the provider can inform the development of preventive and interventional approaches across a range of life domains, motivational and developmental research across the lifespan (e.g. prevention of care-giver burnout), and applied clinical contexts (e.g. therapeutic interventions).

**Trial registration:**

Pre-registration (2023-11-10): https://doi.org/10.17605/OSF.IO/8SPZW, retrospective registration with more details (2024-10-23): https://www.drks.de/DRKS00034287

**Supplementary Information:**

The online version contains supplementary material available at 10.1186/s40359-024-02319-y.

## Background

A substantial body of research has concentrated on the origins of prosocial behaviour (see e.g. the empathy-altruism debate, [1–7]) or on the consequences of receiving social support from the perspective of recipients. Until recently, the question of whether and how the provision of social support can promote *providers’* health was underexplored [8, 9]. This paucity of research may be attributed to the prevalence of evolutionary, social exchange, and equity theories, which focus on the *costs* for support providers (e.g., loss of own resources, [10, 11]). Research on providers of social support frequently reveals negative consequences for the health of the providers. This is especially the case when the support in question is of an intensive nature, such as caregiving. Indeed, there is a substantial body of evidence indicating that caregiving is associated with poor health outcomes for the caregiver (e.g., [12–15]). Only within the last two decades has evidence emerged suggesting that providers of social support may experience *positive psychological and physiological health benefits* [16–20].

The proposed study 2 of the PROSPECT project (Providing Social Support and Health: Conditions and Temporal Dynamics) sets out to investigate the potential conditions for health-promoting effects of providing support for the provider based on self-determination theory.

### Mechanisms connecting support provision to health outcomes

Social support is usually defined as being directed towards a specific individual e.g., as “an exchange of resources between at least two individuals perceived by the provider or the recipient to be intended to enhance the wellbeing of the recipient” ([21], p. 11). It is hence defined by acts of help as compared to the broader terms of *prosocial behaviour* or *beneficence*, which may include acts of kindness towards strangers and the common good as well (e.g., donating money, smiling at others, engaging in environmental cleanup; [22, 23]).

To explain *how* support provision could result in health benefits, at least three interconnected routes have been described – a *physiological*, an *affective*, and a *self-evaluative* route. Evidence for the physiological pathway comes from studies showing associations between greater support provision and reduced stress reactivity at the cardiovascular and endocrine levels, as well as activation of reward-related neural regions of the brain (e.g., blood pressure, heart rate variability, cortisol response, activity in ventral striatum; [18, 24–28]). Providing support may also have an effect on reported and observed positive affect, happiness, flourishing, generativity, and subjective well-being [29–33]. On the self-evaluative route, providing support to others leads to increases in self-esteem and self-worth [18, 34, 35]. Individuals report pride, self-efficacy and purpose in life immediately after supporting others informally (spontaneous helping acts) but also as a consequence of regular formal (organized) support provision, for example through volunteering [1, 36–38].

In contrast, there is substantial evidence indicating adverse effects on health among those providing support. The primary caregivers of ill family members frequently report an increase in depressive symptoms and a negative impact on their subjective and physical well-being [14, 15]. The combination of distinctive characteristics inherent to the caregiver situation – often marked by exposure to a loved one in distress, a non-voluntary arrangement due to financial strains, and a considerable investment of time and energy – result in exceptional scenarios for the provider. Therefore, it has been proposed that caregiving should be investigated under assumptions different from those of day-to-day support provision [39]. Nevertheless, even daily social support has been observed to occasionally elicit physiological stress responses and heightened depressive symptoms among both formal and informal support providers [40–42].

Meta-analyses often conclude that there is a fine line between experiencing support provision as joyful or burdensome [33, 43–45]. To date, research remains inconclusive and contradictory regarding the point at which the provision of social support positively or negatively affects the physiological, affective, self-evaluative and relationship outcomes of the providers. The question, therefore, is: under which conditions does the provision of social support have a beneficial effect on health and well-being and when does it become a burden?

### Proposed conditions for health benefits of providing support

Several moderators have been investigated independently, to better understand why provided support sometimes fails to show health benefits for the provider. For example, volunteering and mental health are only associated at medium volunteering levels (inversely u-shaped, with extreme investments of time becoming detrimental; [46, 47]). Furthermore, if providers feel overcommitted, not reciprocated, and if support is provided for an extended period of time, negative affective reactions are more likely to occur [4, 48, 49]. However, these discussed moderators of the health effects of support do not explicate the basic cognitive processes that must be initiated for support to be beneficial to the provider. Research on the health effects of social support provision has led to the proposition that providing support is healthy for providers only if it is 1) *voluntary* and 2) *effective* [9]. In the PROSPECT trial, we go one step further by suggesting that providing support to others will only result in benefits for the provider if it serves humans’ basic motivational needs. According to self-determination theory, humans strive to fulfil three basic needs – *autonomy, competence*, and *relatedness* [50, 51]. We propose that the likelihood of beneficial effects for the provider should increase if the support occurs under the conditions that it

1. is *freely chosen* (autonomy),
2. elicits providers’ *feelings of impact* on the recipients’ problem (competence),
3. or prompts appreciative *feedback* from the recipient (relatedness).

**Fig. 1:**
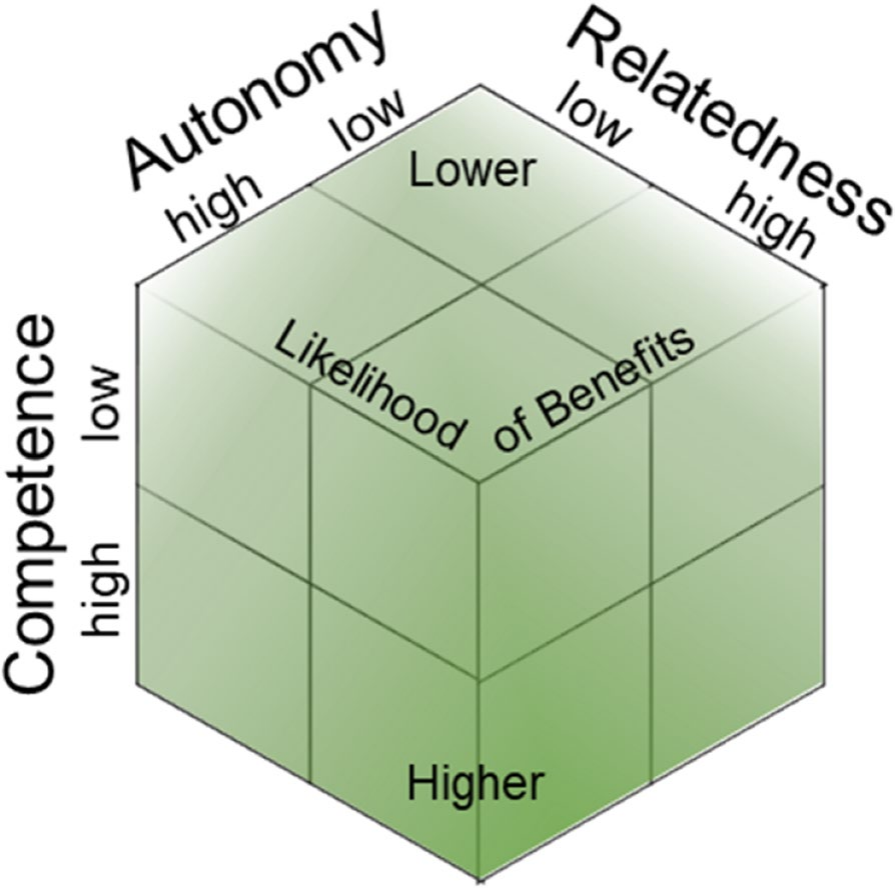
Proposed conditions under which the provision of social support shows benefits for the provider

Figure 1 summarizes this proposition.

### Condition of autonomy

There is ample evidence indicating that choosing to help feels better than feeling obliged to do so. For example, Weinstein and Ryan [52] conducted several studies in which participants who were given the choice of how much money they wanted to give, experienced greater emotional and social well-being (increased positive affect, vitality, self-esteem), whereas participants without a choice did not experience benefits. In addition, neural activity in areas linked to reward processing was found to be higher if participants spent money voluntarily instead of mandatorily [53]. Moreover, even caregivers who felt they had a choice in whether they took responsibility for their loved one’s care, reported significantly less stress, physical strain, and detrimental health than those who did not perceive a choice [54, 55]. Thus, fulfilling the basic human need for autonomy appears critical for deriving benefits from providing support [51]. This condition is not manipulated but set for all experimental groups that provide support in our trial.

### Condition of competence

To date, the condition of *perceived effectiveness* has not been operationalized from the provider perspective – neither as a direct measure nor as an experimental manipulation to test its role in making social support provision beneficial. Some studies in the domain of beneficence examined how altering providers’ perceptions of impact affects outcomes. For instance, Aknin et al. [56] found that if the impact of donations to charity was clearly described, participants exhibited heightened positive affect. Furthermore, providers who set a concrete goal of making someone smile through an act of kindness reported higher levels of happiness than those who formed the abstract goal of making someone happy [57]. In other words, observing a concrete impact on the recipient may enhance the provider's *sense of competence* in providing effective support. At present, the evidence on the condition of competence is limited to affective outcomes following acts of kindness (not concrete support) as opposed to objective physiological health indicators.

### Condition of relatedness

We propose that if the support provided elicits positive *acknowledgment* from the recipient, it will satisfy the providers’ need for relatedness. Previous studies have indicated that support from older parents to middle-aged children must *feel rewarding* to decrease depressive symptoms [41]. Although providers benefitted from any compassionate act they provided to their spouses, acts that were *seen* by the recipient led to greater positive affect for the provider [58]. Also, perceiving that one’s partner *appreciates* the provided support was found to moderate the association between giving support and self-reported physical symptoms, in that higher perceptions of appreciation were associated with fewer physical symptoms [59].

Initial studies of combinations of the three proposed conditions for beneficial support show that participants who reported that their support was *effective* and who felt *socially connected* showed activation of the neural substrates of caregiving [26]. A study by Martela and Ryan [60] even found that a gaming condition, in which participants anonymously donated rice to a food program, led to subjective well-being mediated via feelings of autonomy, competence, and relatedness. However, Ko et al. [61] did not find an ‘acts of kindness’-intervention to increase any of the three basic psychological needs. By specifying the needs as mediators, these studies raise the question of whether needs fulfilment is a *consequence* of helping, or whether fulfilling any or all of the three is a pre-condition (moderator) without which support will not benefit the provider. In a seven-day diary study, providers reported better well-being if they perceived autonomy, felt they had improved the recipient’s situation and experienced gratitude for their helping behaviour [62]. Experimental evidence for these three conditions however remains to be accrued.

### Objectives of study 2

The new experimental paradigm was developed to better understand under which preconditions of psychological needs satisfaction (self-determination theory; [51]) social support provision is beneficial for physiological, affective, self-evaluative, and relationship outcomes in providers. We propose that for positive effects to emerge, providers need to support their dyadic partner autonomously (this condition is not manipulated but present in all support conditions), and to build a sense of *competence* and a sense of *relatedness* in providers. Through the experimental manipulation of *competence* and *relatedness*, the causal conditions under which support giving is beneficial for the provider can be extracted from possible confounding variables, such as differences at baseline (e.g., personality, health) and from possible reversed effects (individuals with greater resources providing more support).

The primary objective of this study is to examine whether support providers who receive feedback on the positive impact of their support on the recipient (competence) and on their dyadic partners' appreciation of the support (relatedness) will demonstrate less pronounced increases and/or faster decreases in their physiological stress reactions, more positive affect, more positive self-evaluative and possibly also relationship evaluative reactions than providers who do not receive such feedback. To achieve this, four conditions will be randomized: 1) No support provision (observation condition = control), 2) autonomous support provision without feedback (autonomous support provision only condition), 3) autonomous support provision with feedback on competence (autonomous support provision + competence condition), and 4) autonomous support provision with feedback on relatedness (autonomous support provision + relatedness condition). The following main hypotheses will be tested:

- H1: The new paradigm of observing the partner person’s alleged stress and pain responses on a computer screen will influence participants’ physiological, affective, self-evaluative, and relational responses in all conditions (manipulation check).
- H2: Physiological, affective, self-evaluative, and relationship responses to observing the partner person's alleged stress and pain levels are higher in participants with higher trait empathy.
- H3: Participants in the support provision conditions will show more beneficial physiological, affective, self-evaluative, and relationship responses than those in the observation conditions that did not get the opportunity to support their partner person.
- H4: Participants who receive feedback on their support provision – in either the competence or relatedness conditions – will exhibit more beneficial physiological, affective, self-evaluative, and relationship responses compared to participants in the observation and autonomous support provision only condition.
- H5: Participants in the competence condition will show more beneficial physiological, affective, self-evaluative, and relationship responses than participants in the relatedness condition.
- H6: Self-reports of competence and relatedness during the experimental manipulation mediate the effects of the condition on physiological, affective, self-evaluative, and relationship responses.

**Fig. 2:**
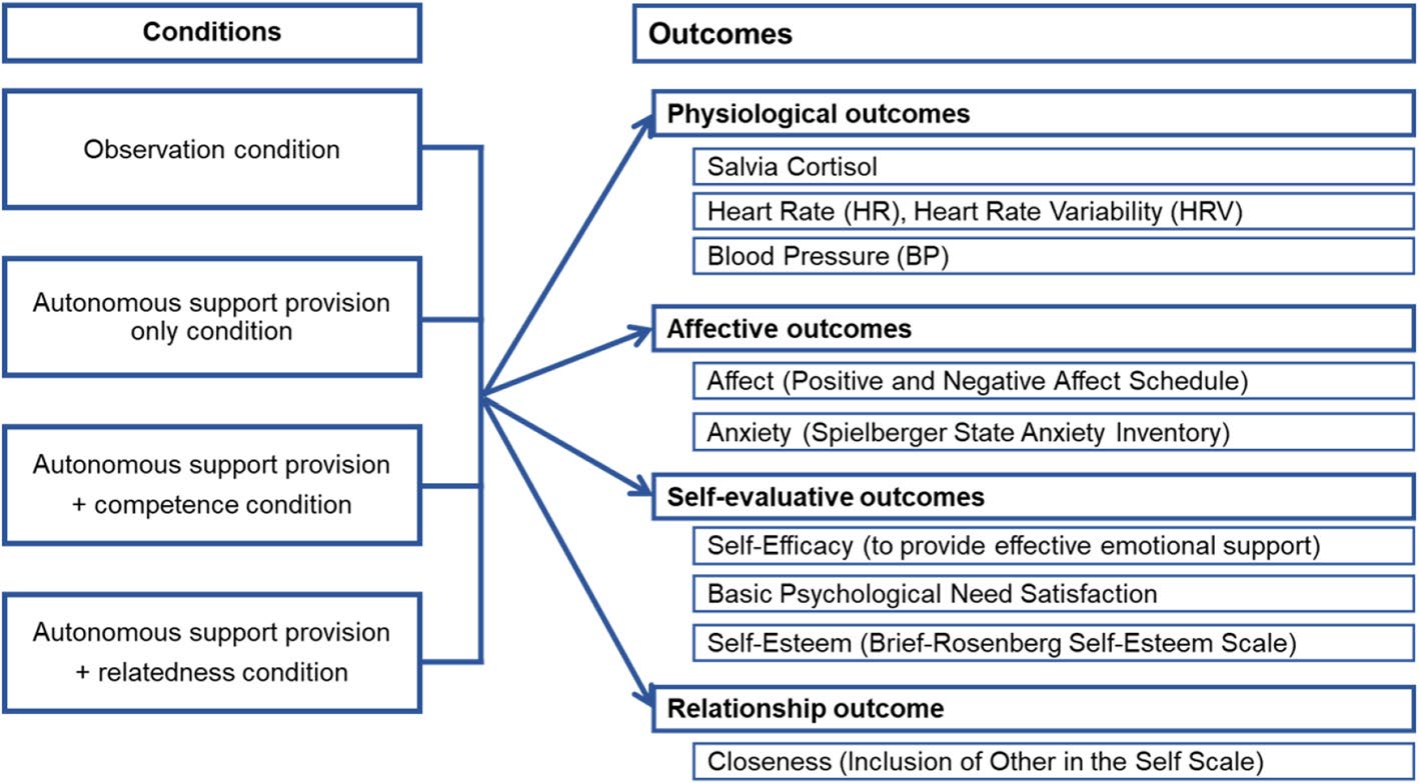
Research model of the experimental conditions and outcomes

The four conditions and the research model are demonstrated in Fig. 2.

In addition to the primary hypotheses, several exploratory analyses will be conducted to investigate inter-individual differences at baseline measurements and their impact on physiological, affective, self-evaluative, and relationship variables in the laboratory setting. For instance, it will be examined whether participants who self-report higher levels of empathy and are tested to confirm this at baseline are expected to demonstrate a greater willingness to swap places with their partner and take over the alleged stress tasks their partner is enduring, as opposed to opting to remain in the supporter role. Furthermore, participants may have differing perceptions of autonomy when providing support, which could potentially impact their physiological, affective, self-evaluative, and relationship responses,  even without deliberate manipulation of autonomy. Differences in relationship satisfaction might moderate processes of support provision and perception. The engagement and emotional tone of the chat messages created in conditions with support provision might relate to providers’ outcomes as well. Additionally, baseline survey data will be utilized to validate the  latest version of the Multifaceted Empathy Test (MET-core-2 ; [63, 64]).

## Methods

### Ethics approval, funding and registration

This study forms part of a larger research project funded by the German Research Foundation (DFG #465093987). This protocol concerns the laboratory part of the study (Study 2). Ethical approval was obtained from the Ethics Committee of the MSB Medical School Berlin (#MSB 2023 − 138) and the study complies with the 1964 Declaration of Helsinki. All participants provide informed consent prior to data collection. Three further studies are part of the PROSPECT research program, one on video observed support provision in dyads (Study 1), the other two on naturalistic support provision in ecological momentary assessments (Study 3 and 4) funded by the National Science Centre Poland (NCN #2020/39-G/HS6/02216). The study was pre-registered at the Open Science Framework (https://osf.io/csqa8/). It was also retrospectively registered with more details at the WHO registry ‘German Register for Clinical Studies’ (DRKS: https://www.drks.de/DRKS00034287).

### Study design

**Fig. 3:**
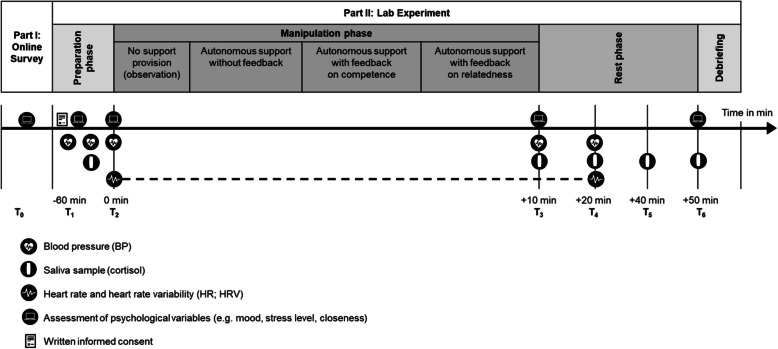
Phases of the study procedure

The laboratory study consists of two parts. In Part I, participants complete the baseline measures in an online survey in a self-chosen environment. In Part II, participants take part in the newly developed experimental paradigm at the MSB Medical School Berlin, Germany. See Fig. 3 for an overview of timepoints and Table 3 for a summary of measures.

Part I – Online Survey (baseline). In the first part of this study, participants individually complete the baseline assessment as an online questionnaire on the survey tool SoSci Survey. This questionnaire assesses inclusion and exclusion criteria for the laboratory part, demographics, as well as a series of self-report questionnaires and an empathy test (see measures section).

Part II – Experimental paradigm with manipulation of conditions. The second part of this study will directly manipulate competence and relatedness as potential conditions for effects on immediate physiological, affective, self-evaluative, and relationship outcomes in an experimental paradigm. Participants are invited into the laboratory together with a close dyadic partner. Both dyadic partners are led to believe that their partner person is enduring several runs of stressful tasks in another room of the laboratory. In three conditions, participants will be able to provide social support to their partner persons via text messages in a computer chat window. In two of these conditions, they receive feedback, either that their messages reduced the stress of their partner person (competence condition) or that their partner person feels related to them because of their messages (relatedness condition).

### Conditions

**Fig. 4:**
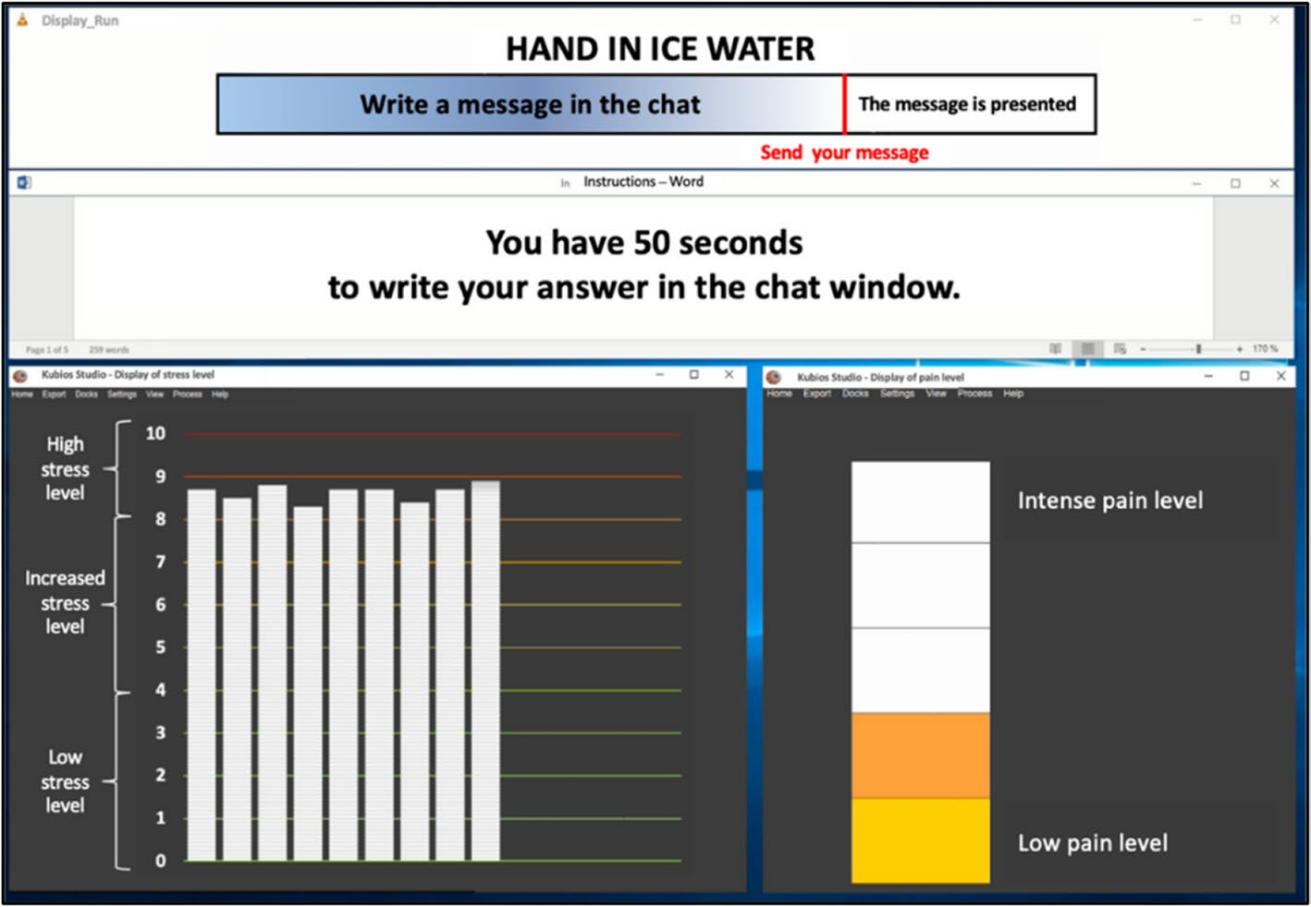
Screenshot of the alleged stress and pain levels their partner persons felt in the support provision conditions (translated to English)

**Fig. 5:**
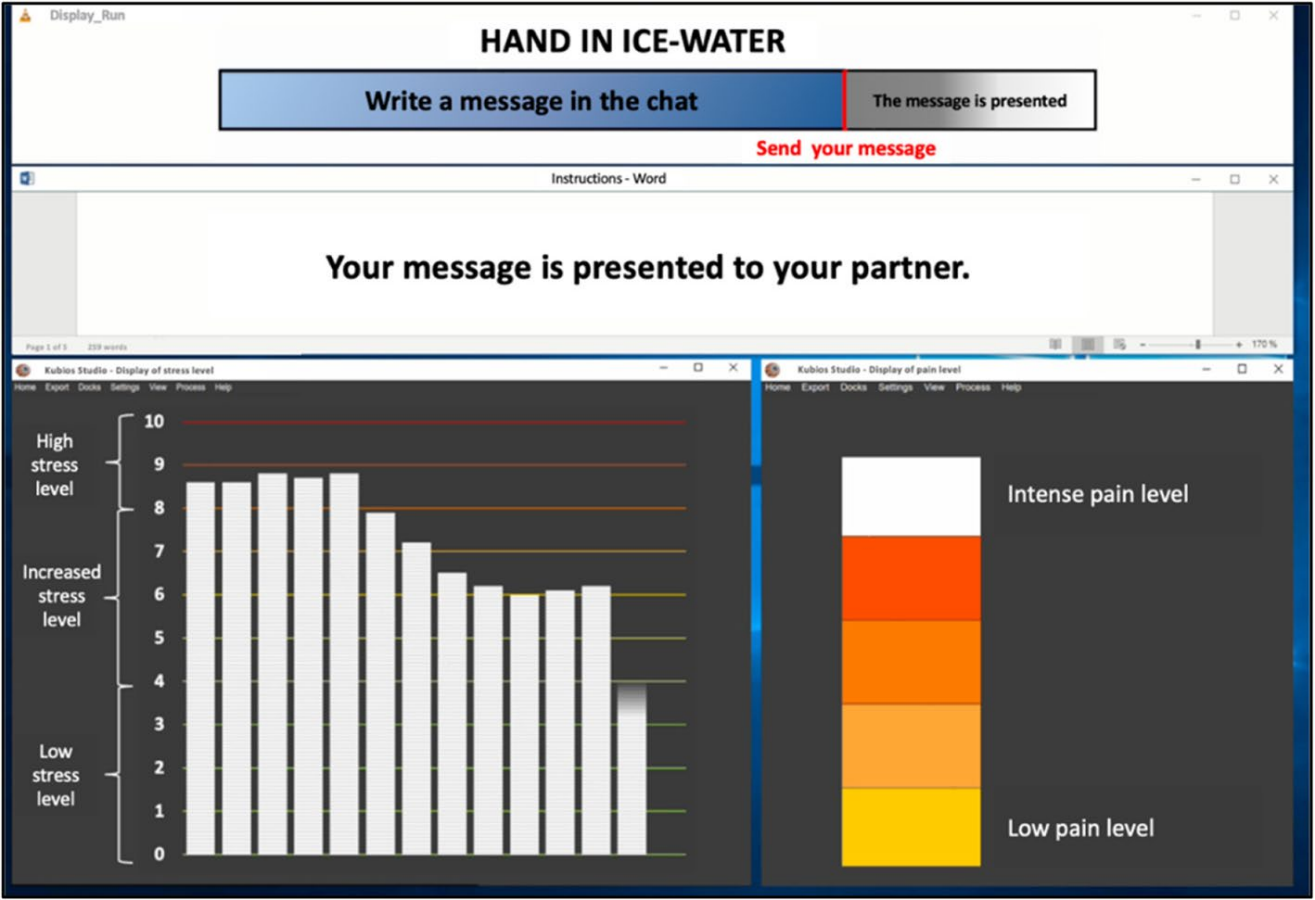
Screenshot of competence condition, in which the stress dropped after participants provided supportive chat messages to their allegedly stressed partner persons (translated to English)

The participants will individually and randomly assign to one of four conditions:

1. No support provision (observation condition = control, see Table 1)
2. Autonomous support provision without feedback (autonomous support provision only condition, see Fig. 4)
3. Autonomous support provision with feedback on competence (autonomous support provision + competence condition, see Fig. 5 and Table 2)
4. Autonomous support provision with feedback on relatedness (autonomous support provision + relatedness condition, see Fig. 4 and Table 2)

Detailed instructions for each condition are available in the study material folder of the German part of the project on OSF (https://osf.io/csqa8).

**Table 1:** Questions for participants in the observation condition (translated to English)

Question nr.	Question
1	How do you think is your partner person feeling at the moment?
2	What do you think is your partner person thinking right now?
3	How do you feel when observing your partner person's stress and pain levels?
4	What do you think when observing your partner person's stress and pain levels?
5	What do you think your partner person will tell you about the stress test afterwards?

**Table 2:**
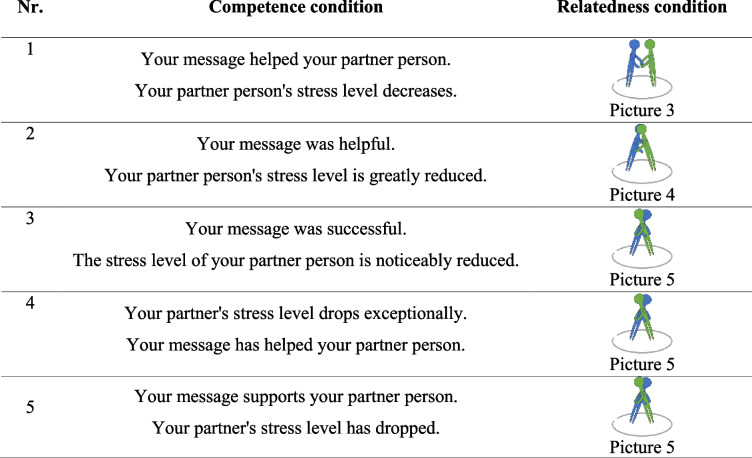
Feedback providers received in the competence and relatedness conditions after they provided supportive chat messages to their allegedly stressed partner persons (translated to English)

Feedback is sent by the research investigators via chat messages

### Randomization

Individual randomization is based on a computer-generated allocation plan. Once a dyad has been scheduled to participate in the second part of the study, each individual is assigned at random to one of the four conditions. For this purpose, the random sequence of conditions is created as a pre-defined list via the online generator (https://www.randomizer.org/).

### Blinding

Participants are blinded about the true purpose of the experiment. All participants are told that the study investigates the effects of *receiving* social support on stress. In the laboratory part, participants are told that a random lottery ticket decides which dyad partner undergoes several runs of a stressful task, while the other dyad partner is observing their stress and pain levels and, depending on the condition, has the opportunity to provide support via chat messages. Each participant is always assigned  the support provider role on their lot. Each participant is made to believe that their partner person is undergoing a stressful task, when in fact neither of them is doing the stressful task. None of the participants know that the study is investigating the effects of providing support on stress and that different conditions are being tested. At the end of the experiment, participants receive a detailed debriefing based on Mills’ procedure for explaining experiments involving deception [65]. The original German version and an English translation of the debriefing conducted by our research assistants can be found at OSF (https://osf.io/8spzw).

### Sample size calculation

Meta-analyses summarizing effects of experimental manipulations of prosocial behaviour on well-being resulted in small- to medium-pooled effects sizes from Cohen’s d = .28 [33] to Pearson’s r = .32 (corresponding to a Cohen’s d = .68; 43). Whereas these effect sizes represent support provision vs. no support provision, we are particularly interested in manipulating and comparing the effects of different conditions under which support is provided. Therefore, we base our power analyses on a small n^2^ of 0.02 for the interaction term of time*condition (Cohen, 1992; effect size f = 0.17). We deem a power of 95% desirable to detect effects not only between the ‘support’ to ‘no support’ group, but also between the groups that provide support under different conditions. With 4 between- and 2 within-subject factors entered into a repeated measures ANOVA, G*power estimates a total *N* = 216 (54 per group) to result in a 95% chance to reject the null hypothesis of no significant interaction term [66]. To allow for attrition due to failed manipulation (e.g., participants provide no support despite randomized to chosen support groups), we plan to recruit 250 participants randomized to four groups (125 dyads).

## Participants

### Eligibility criteria

The following main criteria must be fulfilled for participation in the study: (1) age between 18 and 65 years; (2) informed consent to all aspects of the study; (3) proficiency in German language; and (4) joining the laboratory part of the experiment with another person (romantic partner or friend) with whom they have shared a close relationship for at least six months.

Participants who fulfill the following exclusion criteria will be excluded from participation in the study: (1) psychology students in their 5th or higher bachelor's semester or in the master's program or persons who have completed their psychology studies or (2) with a body mass index (BMI) over 30 [67]; pregnancy or breast feeding [68–70]; heavy smoking (defined as more than 10 cigarettes/day; [69, 71]); substance abuse [67, 69–71]; the presence of cardiovascular disease [67–71] or a neurological, psychiatric or endocrine disorder [67, 68, 70, 71] or the regular intake of hydrocortisol medication [69–71] as these could influence the physiological stress response [72].

### Recruitment

Study participants are recruited by distributing flyers at the Medical School Berlin (MSB), the Free University Berlin (FU) and other universities in Berlin, as well as in the area around the campus of the Medical School Berlin (e.g., neighborhood, cafés, restaurants, stores) and at public events related to psychology or research (e.g., Open Campus Day, Long Night of Science). Study participants are also recruited online through entries on online platforms (e.g., social media).

Individuals who meet the inclusion criteria are asked to give a written informed consent. The research team ensures confidentiality of this data by keeping questionnaire and physiological data and the consent forms in different data storages, to which only research assistants have access. Sensitive data (i.e., phone numbers and email addresses) will be stored only during the data collection period. Once data collection is completed, data will be anonymized and analysed statistically in this form.

### Allowance

Participants enter a lottery for 5 x 50 Euro online shopping vouchers upon completing the online baseline questionnaire and students can choose between entering the lottery or receiving research participation credit. Participants receive 30 Euro per person for taking part in the laboratory part of the trial and students can choose between the 30 Euro or 3-4 hours of research participation credits per person.

### Measures

The baseline assesses all socio-demographic and trait constructs that are not expected to change due to the manipulation in the laboratory part of the study. In the laboratory momentary physiological, affective, self-evaluative, and relationship variables are assessed. The post-manipulation physiological assessments and self-reports repeat some of these momentary states to capture the effects of the four experimental conditions. An overview of the measurement points in time and instruments can be found in the SPIRIT chart in Table 3.

**Table 3:**
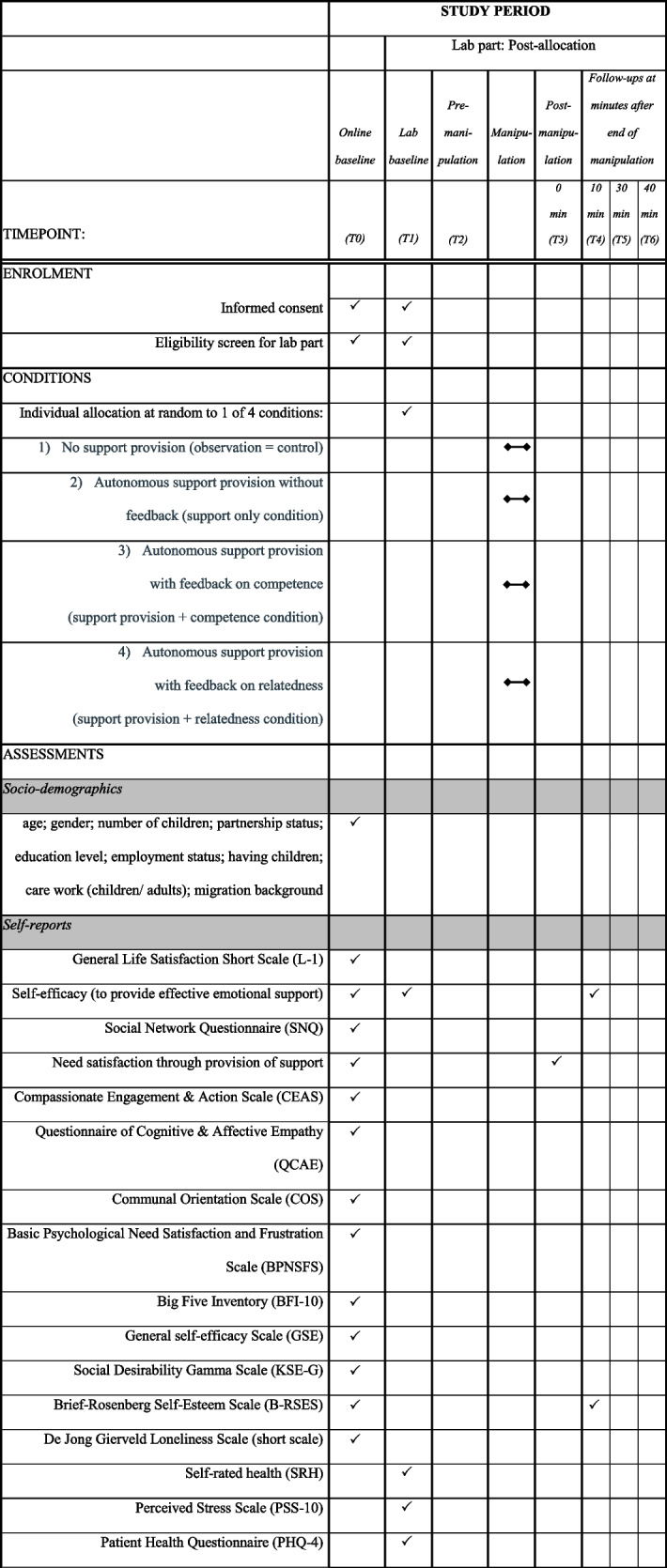

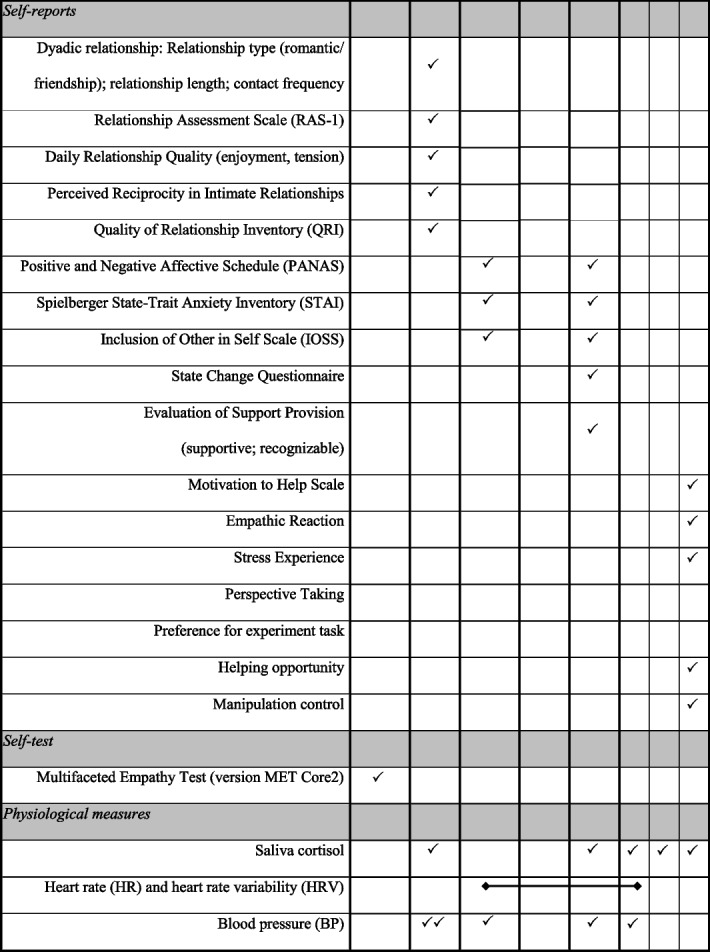
SPIRIT schedule of enrolment, conditions, and assessments according to time points

### Socio-demographics

The following socio-demographic covariates are assessed: gender (male, female, diverse), age, partnership status (single, in an intimate relationship, married, separated, divorced, widowed), education level (German education levels, which can be categorized into three levels of the International Standard Classification of Education^1^ (ISCED, Unesco, 2011), employment status^2^, having children (yes/ no), private care work for children and/or adults and migration background (“Did you or your parents immigrate to Germany?” myself/ one parent/ both parents/ no).

### Self-report questionnaires

Self-report questionnaires are used at baseline, at arrival in the laboratory, directly pre-manipulation, directly post-manipulation and at the last measurements point shortly before debriefing. We describe every instrument shortly and indicate at which measurement points in time it is assessed in Table 3. More details on the measures can be found in the project material on OSF (https://osf.io/8spzw).

#### General life satisfaction short scale

The German version of the 1-item General Life Satisfaction Short Scale (L-1) is used to assess general life satisfaction on a scale ranging from 0 (not at all satisfied) to 10 (completely satisfied) [73].

#### Self-efficacy (to provide effective emotional support)

Following the study by Rossetto, Lannutti and Smith [74], participants self-report their perceived ability to provide emotional support (support-specific perceived self-efficacy) using a three-item efficacy scale adapted from a six-item scale used in previous support research [75]. The questionnaire was translated into German with minor changes in wording. Each item is rated on a 7-point Likert scale from 1 (strongly disagree) to 7 (strongly agree).

#### Social network questionnaire

The Social Network Questionnaire (SNQ) contains items that each represent a dimension of social relationships identified by Barrera [76]. Internal consistency have been described by Krause [77, 78]. The following three scales were selected from the SNQ (and translated to German) to assess the categories of interest for the survey: provided support, enacted support, and negative interactions. Items are scored on a 4-point Likert scale with 1 (never), 2 (once in a while), 3 (fairly often) and 4 (very often). The *provided support* scale measures the amount of emotional, tangible and informational support that respondents provided to others in the past 12 months. The items on the *enacted support* scale measure the estimated amount of emotional, tangible, and informational support received from others in the year prior to the survey. The scale for *negative interactions* measures how often others make excessive demands (1), are critical (2), interfere in the respondent's personal affairs (3) and take advantage of the respondent (4).

#### Need satisfaction through support provision

Based on the event-based scale to assess psychological needs satisfaction by certain events [79], a set of 9 items, translated into German, is used to measure the satisfaction of the basic needs of autonomy, competence and relatedness through the provision of social support with three items each. At baseline, the original instruction “During this event I felt…” was adapted to prompt participants to think of support situations “While providing support in the last month, I felt...”. Directly after the support manipulation in the laboratory, the questionnaire is used again with adapted instructions to measure basic need satisfaction during the experiment “During the task I just completed, I felt ...”. Participants respond on a scale from 1 (not at all) to 5 (very much).

#### Compassionate engagement and action scales

The subscale *Compassion for Others* from the Compassionate Engagement and Action Scales (CEAS; [80]) is used in a translated German version. The first part of the scale reflects compassionate engagement with others and consists of eight items (e.g., “I am motivated to deal with other people's distress and work with them when it occurs.”). The second part of the scale, measuring compassionate actions for others, consists of five items (e.g., “I take action and do things that are helpful to others.”). Each participant is asked to rate the frequency of the statement on a 10-point Likert scale from 1 (never) to 10 (always). The three reverse items were not included in the survey because the original study labeled these items as fillers and excluded them from the final analysis [80, 81].

#### Questionnaire of cognitive and affective empathy

*Cognitive and affective empathy* were measured using the Questionnaire of Cognitive and Affective Empathy (QCAE; [82]) in its German form (items from two previous German translations of the QCAE were used: [83, 84]). The *cognitive empathy* scale (19 items) includes subscales for *perspective taking* (e.g., “I am good at predicting what someone will do.”), and *online simulation* (e.g., “Before criticizing somebody, I try to imagine how I would feel if I was in their place.”). The *affective empathy* scale (12 items) includes subscales for *emotion contagion* (e.g., “It worries me when others are worrying and panicky.”), *proximal responsivity* (e.g., “I get very upset when I see someone cry.”), and *peripheral responsivity* (e.g., “I often get deeply involved with the feelings of a character in a film, play, or novel.”). Each item is rated on a 4-point Likert scale from 1 (strongly disagrees) to 4 (strongly agree) [82].

#### Communal orientation scale

The Communal Orientation Scale (COS; [85]) is a 14-item scale that assesses an individual's perception of the importance of others' needs and feelings in social relationships, as well as their belief in the obligation to assist others and promote their well-being (e.g., “When making a decision, I take other people's needs and feelings into account.”). Respondents answer each item on a 7-point Likert scale ranging from 1 (extremely uncharacteristic of me) to 7 (extremely characteristic of me).

#### Basic psychological need satisfaction

The shortened 12-item version of the Basic Psychological Need Satisfaction and Frustration Scale (BPNSFS; [86]) is used to assess *general basic psychological need satisfaction* in its validated German version [87]. The BPNSFS consists of three subscales: *autonomy*, *relatedness*, and *competence*, each containing satisfaction and frustration items (e.g., satisfaction with autonomy “I feel that my choices express my true self.”). The items are rated on a 5-point Likert scale ranging from 1 (strongly disagree) to 5 (strongly agree).

#### Big five inventory

 The BFI-10 [88] is a short 10-item version of the established Big Five Inventory (BFI; [89]) measuring the Big Five personality traits *extraversion*, *agreeableness*, *conscientiousness*, *neuroticism*, and *openness* with two items per dimension. The items are scored using a 5-point rating scale from 1 (strongly disagree) to 5 (strongly agree).

#### General self-efficacy scale

*General self-efficacy* is assessed with the German five-item short form of the General Self-Efficacy Scale (GSE; e.g., “It is easy for me to stick to my aims and accomplish my goals”; [90, 91]). Items are rated from 1 (strongly disagree) to 4 (strongly agree).

#### Brief rosenberg self-esteem scale

 The German five-item Brief Rosenberg Self-Esteem Scale (B-RSES; [92–94]) assesses *self-esteem* on a 7-point scale from 1 (strongly disagree) to 7 (strongly agree).

#### De Jong Gierveld loneliness scale

The short version of the De Jong Gierveld Loneliness Scale [95] includes *emotional loneliness*, characterized by perceived lack of intimacy in relationships, and *social loneliness*, characterized by perceived lack of quantity of social contact. Answers are rated from 1 (never) to 5 (always).

#### Self-rated health

*Self-rated health* (SHR), also known as self-perceived health, is a subjective evaluation of one's own health status and is assessed with the single item “How is your overall health status?”, which was translated into German. The answering options range from 1 (very good) to 5 (very bad). In comparison with other items for self-assessment of the health status, this item previously showed best construct validity [96].

#### Perceived stress scale

 The German short version of the Perceived Stress Scale (PSS-10; [97, 98]) is used to measure perceived *stress* in the last month before measurement. Cohen [98] found the PSS-10 to be valid and reliable and relatively superior to other versions of this questionnaire (PSS; PSS-4; [98]) in terms of internal consistency and factor structure. The questionnaire consists of ten items, each beginning with “In the last month, how often have you...” and has two subscales: *perceived helplessness* and *perceived self-efficacy*. The answers are given on a 5-point Likert scale ranging from 1 (never) to 5 (very often).

#### Patient health questionnaire for anxiety and depression

The German ultra-brief version of the Patient Health Questionnaire as a screening scale for *anxiety* and *depression* (PHQ-4; [99, 100]) combines the two core criteria for depressive disorders and the two core criteria for generalized anxiety disorder that have also been shown to be good screening items for panic, social anxiety, and posttraumatic stress disorders [101, 102]. The PHQ-4 begins with “Over the last 2 weeks, how often have you been bothered by the following problems?” and continues with different symptoms (e.g., “little interest or pleasure in doing things”). Responses are scored as 0 (not at all), 1 (several days), 2 (more than half the days), or 3 (nearly every day).

#### Relationship questionnaires

Three items assess the *dyadic relationship*, including the type of relationship (friendship/ romantic relationship), the relationship length (in months and years; based on [103]) and the frequency of contact (times per month; including phone calls).

As Fülöp et al. [104] suggested that the single-item Relationship Assessment Scale (RAS-1; [105], German version in [106]) can be used as a representative item for the overall construct of relationship satisfaction, it is used with an adapted wording in this study to measure overall satisfaction with the dyadic relationship “Overall, how satisfied are you with the relationship/ friendship with the person you are here with today?”. The item is rated on the original 5-point Likert scale from 1 (not at all satisfied) to 5 (very satisfied).

The *quality of the interaction* between the dyad partners on the day of the experiment is assessed with two items derived from prior studies on marital interactions [107, 108]. These two items capture the two dimensions of daily relationship quality: pleasure and tension. They were slightly adapted (e.g., “How tense/ enjoyable were your interactions with your partner person today?”). The response options range from 0 (not tense/ enjoyable at all) to 10 (as tense/ enjoyable as they could possibly be).

Following Väänänen et al. [109], the *perceived reciprocity in intimate relationships* and its components are measured according to the recommendations of Antonucci [110] and Hatfield et al. [111]. Focusing on the relationship of the dyads in this study, respondents are asked the following adapted question (translated to German) “In your relationship with this person, which of you gives or receives more support and help; how would you describe your relationship in this respect?” The following options to respond are provided: “I receive support and help more than I give.”, “I give support and help more than I receive.” or “I receive support and help as much as I give.”.

The 25-item Quality of Relationships Inventory (QRI; [112]) measures three aspects of specific relationships: social support, conflict, and depth, which are rated on a 4-point Likert scale, ranging from 1 (not at all) to 4 (very much). In line with the study interest to measure the *quality of the dyadic relationship*, only the items of the two dimensions social support (7 items, e.g., “To what extent could you turn to this person for advice about problems?”) and depth (6 items, e.g., “How significant is this relationship in your life?”) of the validated German QRI [113] are used. In the items, a minor change of the word “person” to “your partner person” was made to ensure that the respondents answered the items with regard to their dyad partner with whom they are undergoing the experiment.

#### Positive and negative affect schedule

*Positive and negative affective states* pre and post support manipulation are assessed with the short version of the Positive and Negative Affect Schedule (PANAS; [114, 115]). The short version of the PANAS consists of five adjectives each to measure positive and negative affect, respectively. The positive subscale describes an enthusiastic, active and alert state. The negative subscale describes the degree of negative tension caused by dejection, anger and anxiety [114, 115]. The German items were taken from the 20-item German adaptation by Breyer and Bluemke [116]. In order to capture the momentary affective state, participants are instructed to answer how they feel “in this moment” on a 5-point Likert scale from 1 (not at all) to 5 (extremely).

#### Spielberger state-trait anxiety inventory

The 10-item German Spielberger State-Trait Anxiety Inventory (STAI; [117, 118]) is used to assess current symptoms of anxiety, e.g., “I feel tense” or “I feel nervous”. Respondents are asked to choose between 1 (not at all) to 8 (very much).

#### Inclusion of other in the self scale

The Inclusion of Other in the Self Scale (IOS; [119]) is used to measure perceived *closeness* of the dyad partners pre and post manipulation. This single item is a pictorial measure of closeness, in which participants are asked to choose one of seven increasingly overlapping circles, each representing a greater degree of closeness. Instructions were translated into German.

#### State change questionnaire

 Based on the study by Løseth et al. [120], participants complete the State Change Questionnaire to capture manipulation-related changes in participants’ overall sense of subjective state. The two items assess whether participants feel better or worse after the manipulation and are answered on a visual sliding scale from 0 (not at all) to 100 (very much) [120]. The item time frame was adapted to “To what extent do you feel better/ worse than the last time you were asked about your mood?” and translated into German.

#### Evaluation of support provision

Based on Gallagher et al. [103], one item is used to capture the *provider’s own evaluation of support* (“How supportive do you think you were to the person being tested?”). Furthermore, two additional items were created in German language: “How helpful do you think you were to your partner person during the stress test?” and “How clearly recognizable was your support for your partner person during the stress test?” to measure the *provider’s own evaluations of recognizability of the provision of support*. Participants can respond from 1 (not at all) to 6 (extremely) plus one additional option 0 (“I have not written any support messages today”; for the observation condition).

#### Empathic reaction

Participants’ *empathic reaction* to their observation of their dyad partner's stress during the manipulation (“How well do the following adjectives describe how you felt emotionally when you observed your partner person's stress?”) is measured using six adjectives from the 23-item Emotional Response Questionnaire (ERQ; [121]). These adjectives of empathic concern (sympathetic, moved, compassionate, tender, warm and softhearted) can be split into the two components sympathy and tenderness [122, 123]. The items were translated into German and are rated on a scale from 1 (does not apply at all) to 5 (fully applies).

#### Motivation to help scale

Following Stehr [124], it is assessed whether participants experienced their support provision as autonomous or controlled with the German six-item version of the *Motivation to Help Scale* (original version by Weinstein & Ryan, [52]). Three items assess the extent to which participants experienced their support provision during the stress test as autonomous (e.g., “Because I thought it was important to act in this way.”) or controlled (e.g., “Because I’d feel like a bad person if I didn’t.”). Each item is rated on a scale from 1 (not at all true) to 5 (very true), while the control condition can choose “I did not write any support messages today”.

#### Prosocial behaviour

*Prosocial behaviour* is assessed with one item based on Batson et al.’s [125] prosocial behaviour experiments asking participants whether they would want to replace their partner for the second phase of the alleged six runs of the stress tests post-manipulation. Possible answers are “Yes, I would like to replace my partner person.” or “No, I would like to continue as the observer or supporter.”.

### Self-test

#### Multifaceted empathy test

Only at baseline, the cognitive and emotional aspects of empathic functioning are tested multidimensionally by the new version of the Multifaceted Empathy Test (version MET-core-2; [63, 64]). The MET-core-2 consists of a series of photographs, most of which depict individuals in emotionally charged situations. To assess cognitive empathy, subjects are asked to infer the mental states of the individuals shown in the photographs from a list of four options (higher numbers of correct answers are interpreted as higher cognitive empathy). To assess emotional empathy, subjects rate their emotional reaction in response to the pictures on a 9-point scale (emotional empathy as mean score of emotional reaction to the pictures). This test is not repeated as it will be used as a trait predictor for support provision measures.

### Covariates

#### Social desirability gamma scale

The German Social Desirability Gamma Scale (KSE-G; [126]) measures two aspects of the gamma factor of social desirability, the exaggeration of positive qualities and the minimization of negative qualities, with three items each. The 5-point rating scale ranges from 1 (doesn’t apply at all) to 5 (applies completely).

#### Initiative for study participation

As previous studies have shown that motivation has an impact on whether participants pay close attention to instructions and that motivation can affect study results [127, 128], a single item was developed to ask participants about their *initiative for study participation*: “Did you bring your partner person to this study or did your partner person bring you?”. The possible answers are: “I brought my partner person to this study”, “My partner person brought me to this study” or “We both became aware of the study and jointly agreed to take part in it”.

#### Preference for experimental task

The *preference for experimental task* (stress task vs. observer/ support provider) is assessed as a confounder with “Before starting the experiment, did you have a preference for either condition?” with the options “I was satisfied in my condition.”, “I did not care.”, and “I would have preferred to go through the stress test.”. We assume that the preference for one of the tasks of the experiment could affect the motivation to complete the experiment conscientiously (e.g., [127]).

### Manipulation checks

#### Stress experience

The participants' *individual perception of stress* during different phases of the test situation is assessed by four items developed for this study. These items are intended to show whether and to what extent participants are stressed by observing the stress and pain level of their dyad partner and at which phases of the experiment they feel stressed. Participants rate their perceived stress experience when observing their dyad partner's level of stress and pain during the stress test (1), when writing their support message to their dyad partner (2), when sending their support message to their dyad partner (3) and when receiving feedback via the chat on a scale (4; only in conditions in which feedback is received) on a visual sliding scale from 0 (not at all) to 100 (very much). Another response option is “I did not write any support messages today.” for participants in the control condition.

#### Perspective taking

*Perspective taking* is assessed as a manipulation check with three items from Pahl and Bauer [129] and the term “speaker” was replaced by “partner person”, “I imagined how my partner person is feeling right now.”, “I imagined how I would feel in the situation.”, and ”I put myself in my partner person's shoes.” with options on 9-point Likert scales ranging 1 (not at all) to 9 (very much).

#### Credibility

*Credibility* was assessed with two items on a semantic differential answering format “The experimental situation made the following impression on me…” from 1 (artificial) to 10 (real), and from 1 (incredible) to 10 (credible) and one item to assess whether participants in the support provision conditions perceived thankfulness from the alleged recipients “My partner person was grateful for my support during the stress test” with answers ranging from 1 (not at all) to 10 (very much), and one option for the control group “I did not write any support messages today.”.

### Physiological measures

During the laboratory experiment, three psychological parameters (cortisol, heart rate and heart rate variability, blood pressure) are taken as indicators of stress reactivity (e.g., [130–132]). All assessment points can be found in Table 3.

#### Saliva cortisol

 Saliva cortisol secretion, as a physiological response to stress, involves the release of a glucocorticoid hormone in humans that reflects the adaptation of the hypothalamic-pituitary-adrenal (HPA) axis to stressors [68]. The assessment of cortisol levels in saliva is a reliable, repeatable, safe, and non-invasive method for assessing stress reactions [131, 133, 134]. Saliva cortisol is assessed by means of five non-invasive saliva samples during the laboratory phase of the study at baseline, pre-manipulation, directly post-manipulation (0 minutes), 10 minutes follow-up, 30 minutes follow-up and 40 minutes follow-up (see Fig. 3; use of Salivette® Cortisol, Sarstedt).

#### Heart rate and heart rate variability

During the manipulation phase, the heart rate (HR) and heart rate variability (HRV) is recorded using the Polar H10 chest belt (Polar Electro UK Ltd). Data are stored via Bluetooth with the Kubios software [135] on mobile devices and the Kubios software is used on laptops for data analysis.

#### Blood pressure

Systolic and diastolic blood pressure is assessed using an upper-arm blood pressure monitor (Beurer BM58) during the laboratory phase of the study at baseline (twice, directly at arrival and after the acclimatization phase, about 30 min after arrival), pre-manipulation, directly post-manipulation (0 minutes) and 10 minutes follow-up.

### Planned analyses

Analyses of variance and linear mixed models between conditions and pre- and post-assessments will be performed on self-reported self-evaluative and affective as well as, physiological data and relationship variables. Of particular interest will be changes in positive and negative affect and ratings of competence and relatedness depending on the condition.

Data from participants who do not complete the full manipulation (that drop-out before the post-assessment) or show suspicion (e.g., enter trick questions into the chat boxes in order to check, whether they chat with their partners) will be analysed separately in per protocol and intention-to-treat analyses. No outliers are expected in the self-report data. In the event that participants fail to comply with the inclusion criteria for cortisol assessment (no strenuous exercise, food, alcohol, tooth brushing, smoking, caffeine, or cortisol affecting medication prior to the experiment), their cortisol data may have to be excluded post-hoc. Women who take oral contraceptives are included in the sample. For physiological data (cortisol, heart rate, heart rate variability, blood pressure) we consider truncation at 2 SD from the sample mean if outliers occur. Missing data will be handled via Full Information Maximum Likelihood (FIML).

In exploratory qualitative content analyses, we will investigate type and amount of support messages written into the computer chat boxes by participants in the three support provision conditions.

## Discussion

The current research evidence suggests that individuals who engage in prosocial behaviour tend to report higher levels of subjective well-being [33, 43, 136], greater life satisfaction [137], and a reduced mortality risk [8] among other benefits. Conversely, research has repeatedly demonstrated that, for instance, caregiving can result in burnout and health limitations. The new experimental paradigm described in this protocol, bases on self-determination theory [50], and employs a controlled experimental design to manipulate the conditions of providing social support in a laboratory setting to assess its impact on a range of outcomes, including physiological, affective, self-evaluative, and relationship indicators in dyads.

The objective of the paradigm is to examine the circumstances under which providing social support (via chat messages) to a close social network member (in another room) has beneficial effects for the provider. The observation condition serves as the control condition, in which participants are presented with an alleged stress and pain response by their partner person (on a computer screen), who allegedly endures stress tasks in another room. This observation condition is compared to a condition in which participants are given the opportunity to send supportive chat messages to their allegedly stressed partner person, without receiving any feedback on their supportive efforts (support provision only condition). In order to manipulate perceptions of competence in providers, the third condition (support provision with feedback on competence condition) manipulates partner persons' stress responses shown to providers in a way that after each supportive message a decrease in partner persons’ stress response is shown. The fourth condition manipulates perceived relatedness by showing hugging icons, allegedly sent by the partner person, in response to supportive messages by the provider (support provision with feedback on relatedness condition). The third basic psychological need, autonomy, was not manipulated. However, in each support provision condition, it was highlighted that writing supportive messages is voluntary.

By considering the beneficial effects on relatedness and competence, in addition to autonomy, our study aims to investigate basic psychological processes set into motion by providing social support. This basic knowledge might be used to explain more applied phenomena in everyday support provision. For example, prolonged caregiving for chronically or terminally ill patients has displayed serious detrimental effects but simultaneously beneficial effects on caregivers’ health and longevity [17]. Whereas studies that show detrimental effects of caregiving rely on self-reports from care providers regarding the amount of caregiving [138, 139], the surprisingly positive effect of caregiving on longevity by Brown et al. [17] is based on hours of care *reported by recipients*. Caregivers often feel *obliged* to care for loved ones and give prolonged care they *perceive* as ineffective, resulting in higher levels of stress [39, 140]. Nevertheless, if the level of care provided is acknowledged by the recipient, the efforts of the caregiver may still facilitate the formation of a sense of relatedness [141]. This way, appreciation by recipients may help turn even obligatory, less effective support provision into opportunities to fulfil the basic psychological need for relatedness. As discerning the underlying condition of such complex processes in everyday life is challenging, our paradigm provides a means of manipulating feedback for support provision in a controlled laboratory setting. The results of the described experimental approach will be of particular value when synthesized with measurements taken under natural conditions in the ecological assessment design of the same project (Study 3 and 4, see OSF: https://osf.io/8spzw). This will provide insights into how helping behaviours may impact the helpers themselves, either positively or negatively. Furthermore, these findings can provide insight into how supporting others can have detrimental or beneficial effects on the providers of support. They can also indicate whether the benefits of support provision depend on the satisfaction of basic motivational needs. This can inform the development of preventive and interventional approaches in various areas of life, including romantic and peer relationships (e.g., help acknowledgment in partnerships, support provided to co-workers), motivational and developmental research over the life-span (e.g., prevention of caregiver burnout, volunteering in middle age and active aging, motives for helping), and the applied clinical context (e.g. therapeutic relations and interventions).

## Supplementary Information

Supplementary Material 1.

## Data Availability

Materials of the study procedure (PowerPoint presentations explaining the experiment to the test subjects for each condition, Text for debriefing) and details on the scales and measures can be found in the project material on OSF (https://osf.io/8spzw). Data sharing is not applicable to this article as data collection is still ongoing. The anonymised data supporting the findings of future publications from the PROSPECT study will be made available as open data via the Open Science Framework project page: 10.17605/OSF.IO/CSQA8.

## Footnotes

1 Education levels: ‘advanced’ (university degree or high school diploma qualifying for university admission), ‘intermediate’ (middle/secondary school diploma), and ‘basic’ (secondary school diploma, no diploma, at most 9 years school education)

2 Employment levels: full/part-time employed (including ‘mini-jobs’); apprentice/trainee; working on a ‘one-euro job’-basis; unemployed / looking for work; in partial retirement; retired; school student at a general education school; specialized student; university student; housewife/ househusband; voluntary social or ecological year; federal voluntary service; or other to be specified in open text field
